# Supplementary material for: Dysregulated Antibody, Natural Killer Cell and Immune Mediator Profiles in Autoimmune Thyroid Diseases
Source: Cells. 2020 Mar 9;9(3):665. doi: 10.3390/cells9030665 (PMC7140647; doi:10.3390/cells9030665)
Supplement: Supplementary file 1 [file cells-09-00665-s001.zip › cells-730774-Supplementary Materials/cells-730774-Supplementary Materials-figures.docx]

**Supplementary Materials:** The following are available online at www.mdpi.com/xxx/s1,


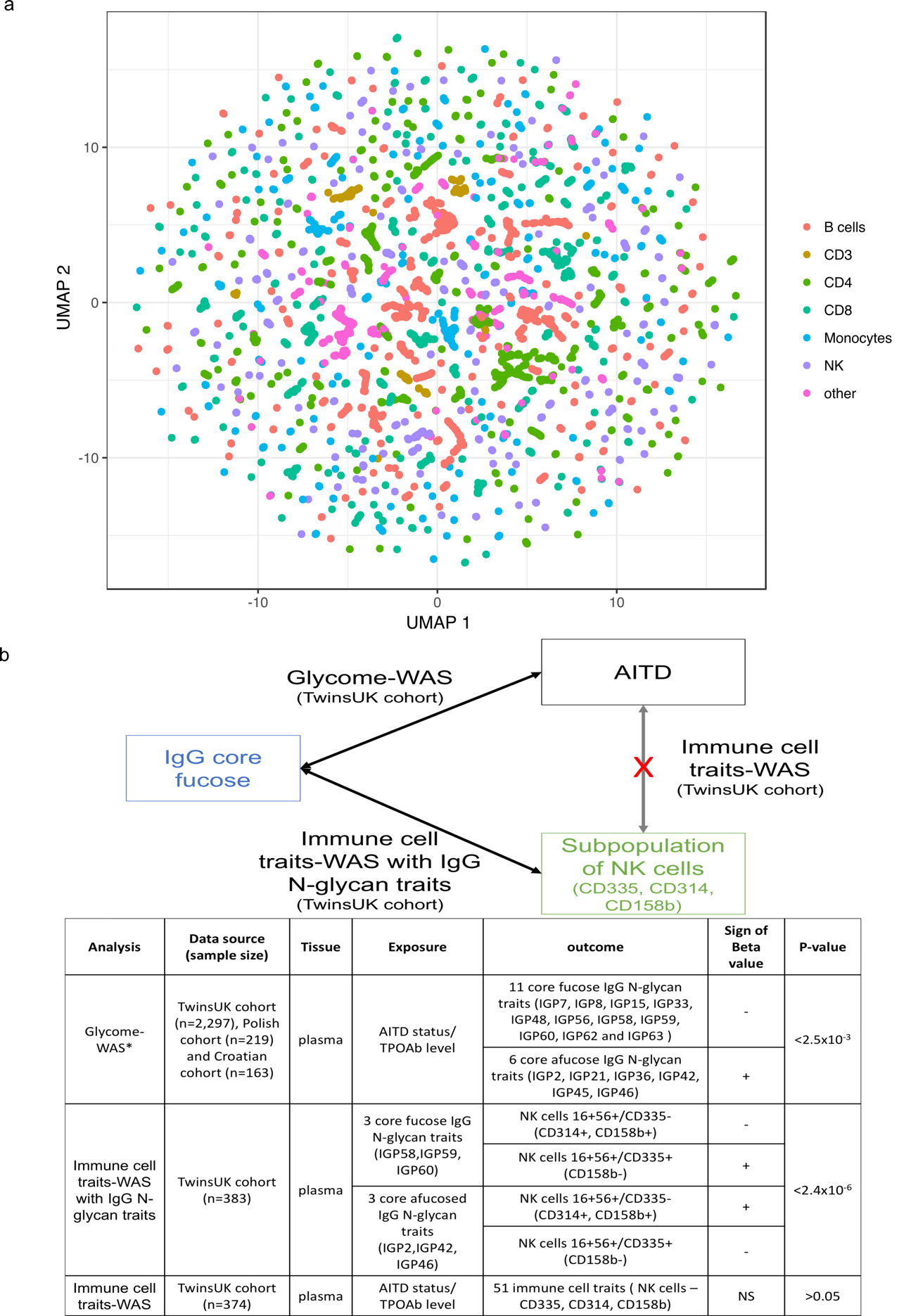


Figure S1: Immune cell traits and AITD status. (a) Immune cell traits were arranged in two dimensions based on the similarity of their quantification profiles by the dimensionality reduction technique UMAP [72] using R package umapr [73]. Some clusters that emerge spontaneously can be associated with specific immune cell types (colors). (b) Overview of associations observed between IgG core-fucose, a subpopulation of NK cells and AITD status in the TwinsUK cohort. *Glycome-wide association studies of AITD and TPOAb levels were previously performed [4].


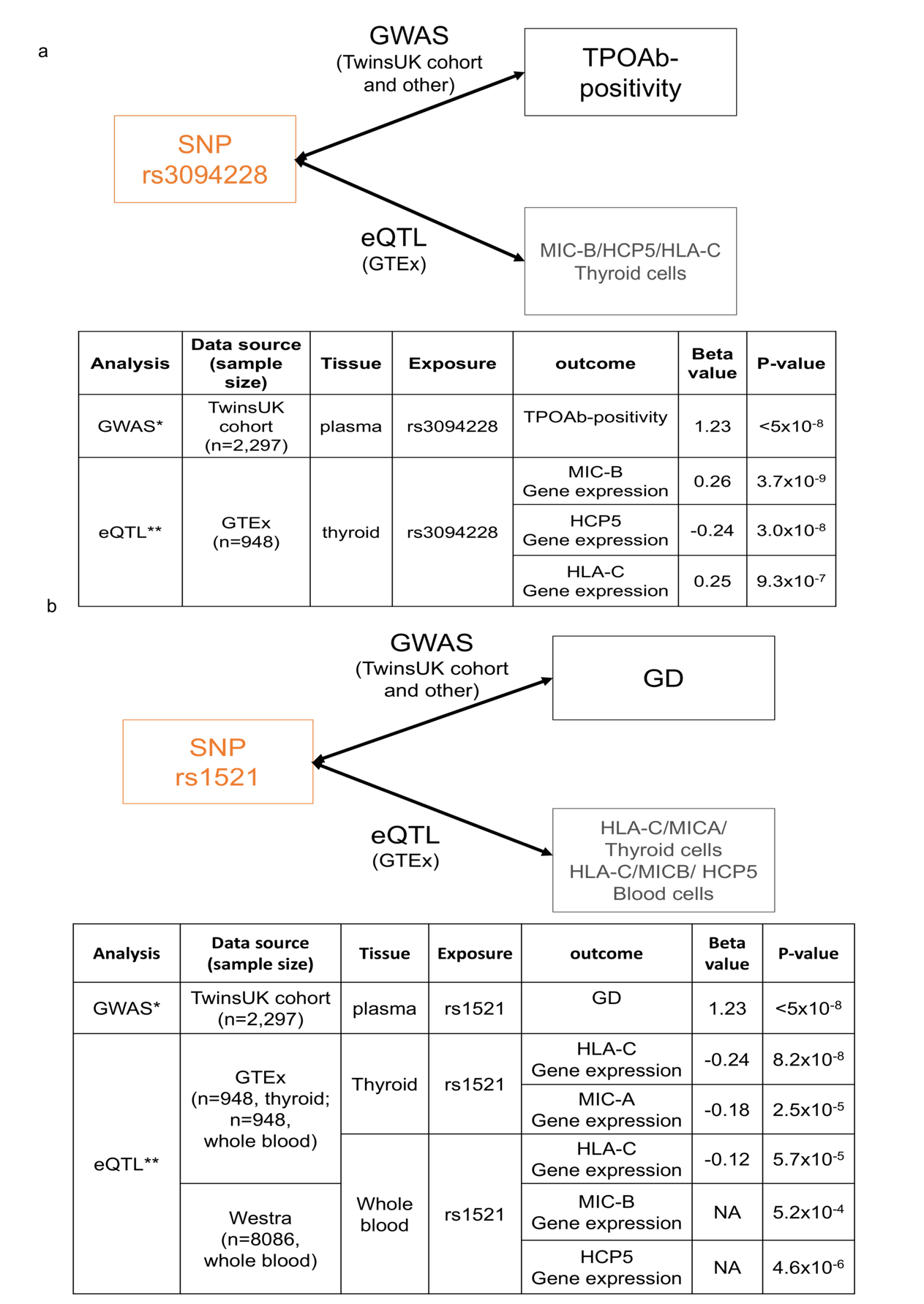


Figure S2. Overview of associations between AITD-SNP and eQTL in thyroid and blood cells. *Genome-wide association studies of AITD and TPOAb-positivity were previously performed, and the findings are available via GWAS catalog [30] whereas **eQTLs come from GTEx project [34] and Westra and al. [35]. (a) Associations between AITD-SNP and eQTL in thyroid and blood cells for the genetic variant rs3094228. (b) Associations between AITD-SNP and eQTL in thyroid and blood cells for the genetic variant rs1521.


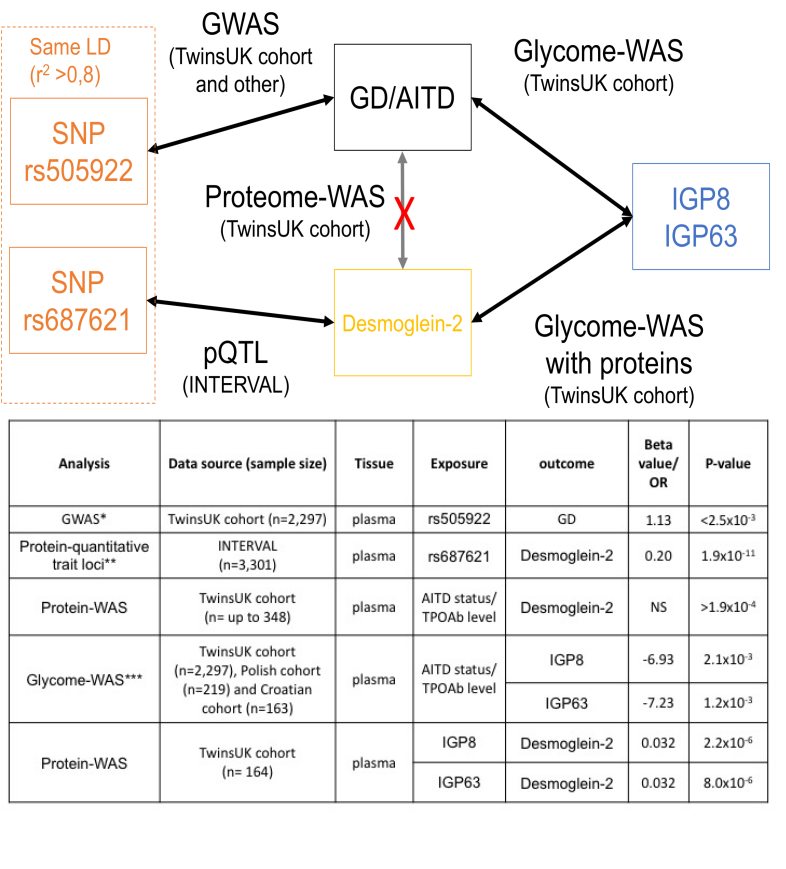


Figure S3. Overview of multi-omic findings associated with Desmoglein-2 in individuals with AITD status and general population. We highlighted a locus with high LD having SNPs and two IgG glycan traits that are both associated with GD and the abundance of secreted plasma Desmoglein-2 in plasma. However, no direct association of AITD status with the abundance of secreted plasma Desmoglein-2. We previously performed glycome-wide association studies of AITD and TPOAb levels [4]. Genome-wide association studies of AITD and TPOAb-positivity were previously performed, and the findings are available via GWAS catalog [30] whereas pQTLs come from INTERVAL project[31]. IGP8 = the percentage of FA2[3]G1 glycan in total IgG glycans. IGP63 = The percentage of fucosylation (without bisecting GlcNAc) of agalactosylated structures.
