# Supplementary material for: Dysregulated Antibody, Natural Killer Cell and Immune Mediator Profiles in Autoimmune Thyroid Diseases
Source: Cells. 2020 Mar 9;9(3):665. doi: 10.3390/cells9030665 (PMC7140647; doi:10.3390/cells9030665)
Supplement: Supplementary file 1 [file cells-09-00665-s001.zip › cells-730774-Supplementary Materials/cells-730774-Supplementary Materials-figures.pdf]

Supplementary Materials: The following are available online at www.mdpi.com/xxx/s1,

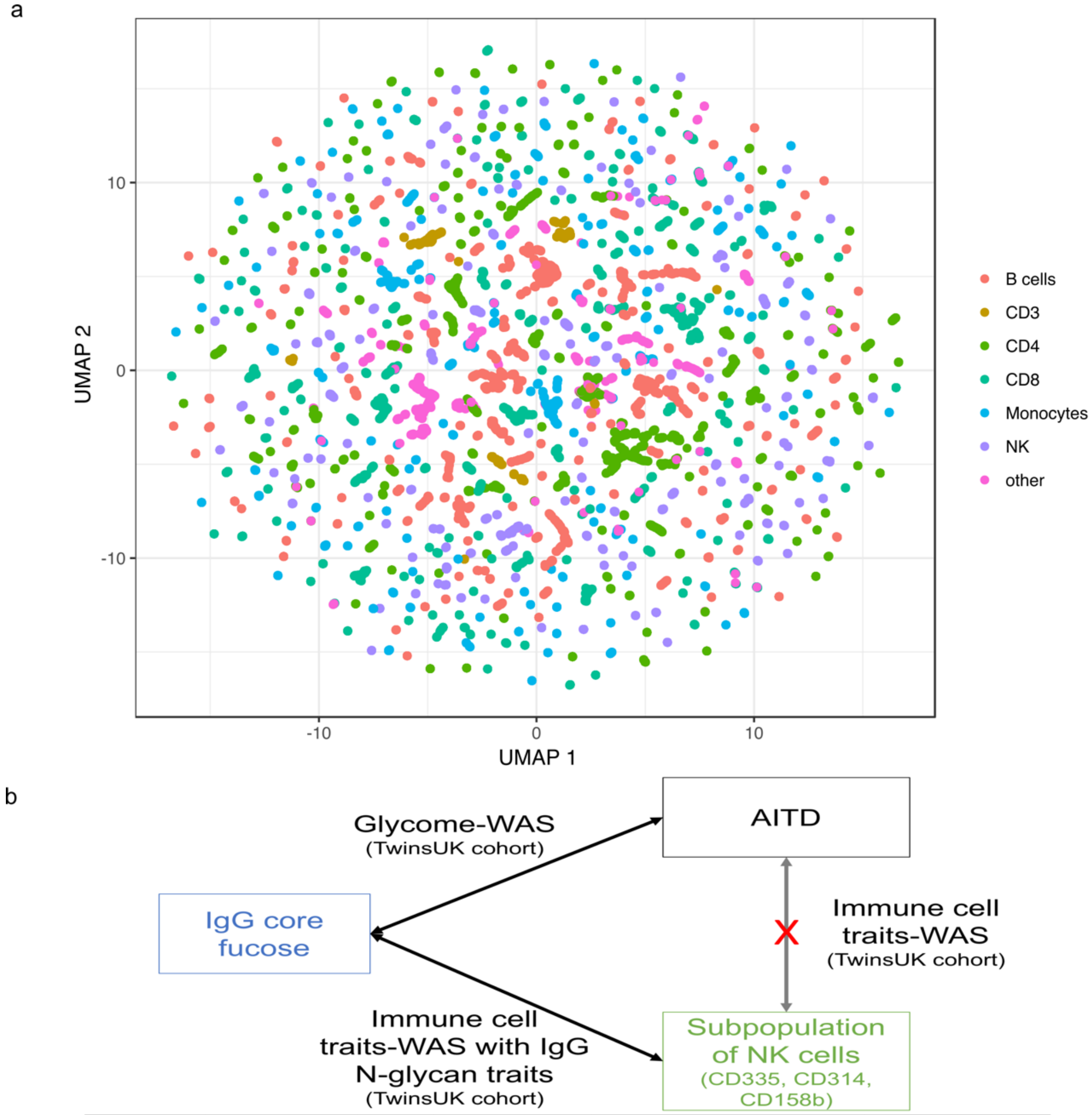

Analysis	Data source (sample size)	Tissue	Exposure	outcome	Sign of Beta value	P-value
Glycome-WAS*	TwinsUK cohort (n=2,297), Polish cohort (n=219) and Croatian cohort (n=163)	plasma	AITD status/ TPOAb level	11 core fucose IgG N-glycan traits (IGP7, IGP8, IGP15, IGP33, IGP48, IGP56, IGP58, IGP59, IGP60, IGP62 and IGP63)	-	<2.5x10^{-3}
				6 core afucose IgG N-glycan traits (IGP2, IGP21, IGP36, IGP42, IGP45, IGP46)	+	
Immune cell traits-WAS with IgG N-glycan traits	TwinsUK cohort (n=383)	plasma	3 core fucose IgG N-glycan traits (IGP58,IGP59, IGP60)	NK cells 16+56+/CD335- (CD314+, CD158b+)	-	<2.4x10^{-6}
				NK cells 16+56+/CD335+ (CD158b-)	+	
			3 core afucosed IgG N-glycan traits (IGP2,IGP42, IGP46)	NK cells 16+56+/CD335- (CD314+, CD158b+)	+	
				NK cells 16+56+/CD335+ (CD158b-)	-	
Immune cell traits-WAS	TwinsUK cohort (n=374)	plasma	AITD status/ TPOAb level	51 immune cell traits (NK cells – CD335, CD314, CD158b)	NS	>0.05

Figure S1: Immune cell traits and AITD status. (a) Immune cell traits were arranged in two dimensions based on the similarity of their quantification profiles by the dimensionality reduction technique UMAP [72] using R

package umapr [73]. Some clusters that emerge spontaneously can be associated with specific immune cell types (colors). (b) Overview of associations observed between IgG core-fucose, a subpopulation of NK cells and AITD status in the TwinsUK cohort. *Glycome-wide association studies of AITD and TPOAb levels were previously performed [4].

a

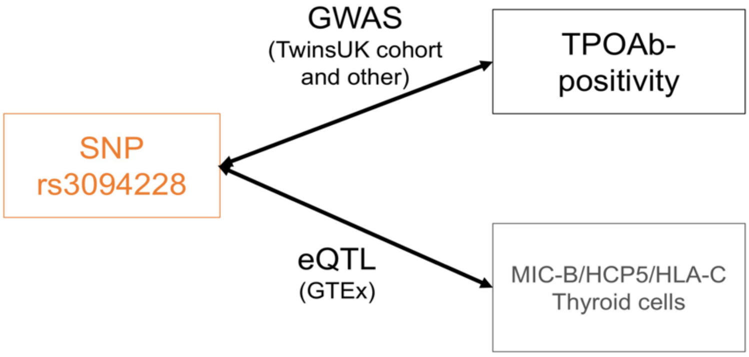

Analysis	Data source (sample size)	Tissue	Exposure	outcome	Beta value	P-value
GWAS*	TwinsUK cohort (n=2,297)	plasma	rs3094228	TPOAb-positivity	1.23	$<5x10^{-8}$
eQTL**	GTEx (n=948)	thyroid	rs3094228	MIC-B Gene expression	0.26	$3.7x10^{-9}$
				HCP5 Gene expression	-0.24	$3.0x10^{-8}$
				HLA-C Gene expression	0.25	$9.3x10^{-7}$

b

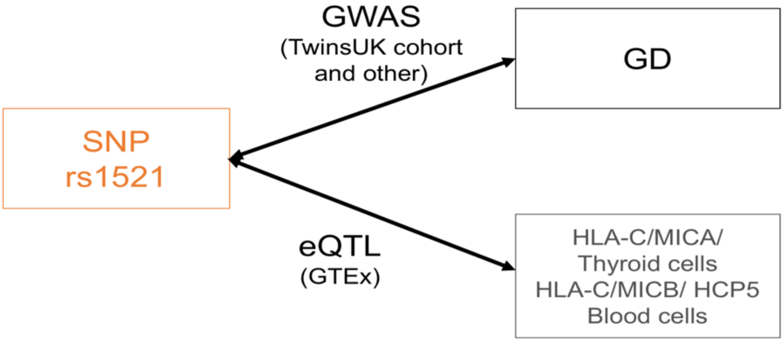

Analysis	Data source (sample size)	Tissue	Exposure	outcome	Beta value	P-value
GWAS*	TwinsUK cohort (n=2,297)	plasma	rs1521	GD	1.23	$<5x10^{-8}$
eQTL**	GTEx (n=948, thyroid; n=948, whole blood)	Thyroid	rs1521	HLA-C Gene expression	-0.24	$8.2x10^{-8}$
				MIC-A Gene expression	-0.18	$2.5x10^{-5}$
		Whole blood	rs1521	HLA-C Gene expression	-0.12	$5.7x10^{-5}$
	Westra (n=8086, whole blood)			MIC-B Gene expression	NA	$5.2x10^{-4}$
				HCP5 Gene expression	NA	$4.6x10^{-6}$

Figure S2. Overview of associations between AITD-SNP and eQTL in thyroid and blood cells. *Genome-wide association studies of AITD and TPOAb-positivity were previously performed, and the findings are available via GWAS catalog [30] whereas **eQTLs come from GTEx project [34] and Westra and al. [35]. (a) Associations

between AITD-SNP and eQTL in thyroid and blood cells for the genetic variant rs3094228. (b) Associations between AITD-SNP and eQTL in thyroid and blood cells for the genetic variant rs1521.

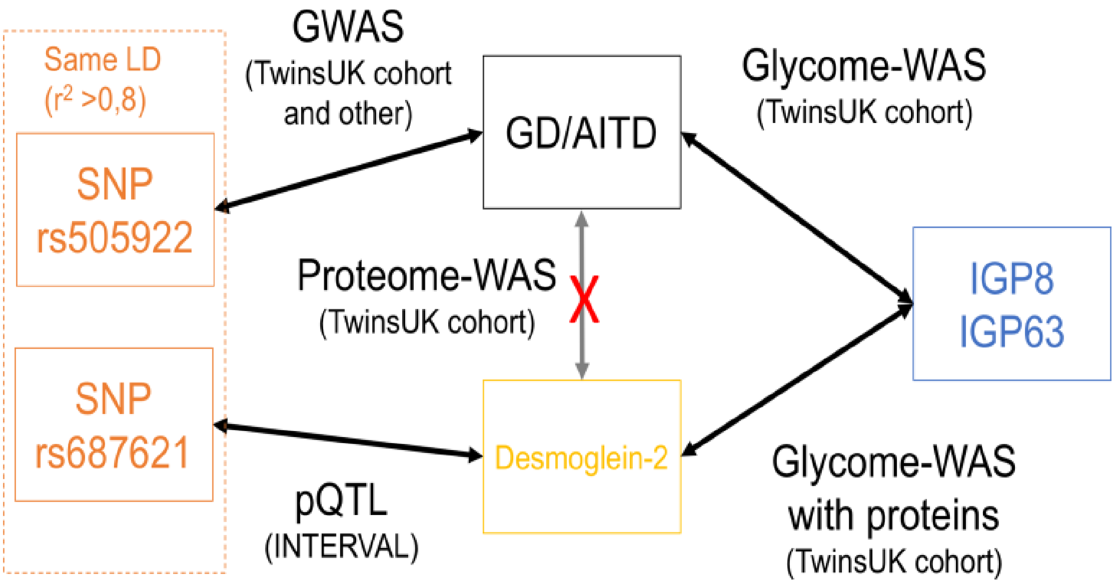

Analysis	Data source (sample size)	Tissue	Exposure	outcome	Beta value/ OR	P-value
GWAS*	TwinsUK cohort (n=2,297)	plasma	rs505922	GD	1.13	$<2.5\text{x}10^{-3}$
Protein-quantitative trait loci**	INTERVAL (n=3,301)	plasma	rs687621	Desmoglein-2	0.20	$1.9\text{x}10^{-11}$
Protein-WAS	TwinsUK cohort (n= up to 348)	plasma	AITD status/ TPOAb level	Desmoglein-2	NS	$>1.9\text{x}10^{-4}$
Glycome-WAS***	TwinsUK cohort (n=2,297), Polish cohort (n=219) and Croatian cohort (n=163)	plasma	AITD status/ TPOAb level	IGP8	-6.93	$2.1\text{x}10^{-3}$
				IGP63	-7.23	$1.2\text{x}10^{-3}$
Protein-WAS	TwinsUK cohort (n= 164)	plasma	IGP8	Desmoglein-2	0.032	$2.2\text{x}10^{-6}$
			IGP63	Desmoglein-2	0.032	$8.0\text{x}10^{-6}$

Figure S3. Overview of multi-omic findings associated with Desmoglein-2 in individuals with AITD status and general population. We highlighted a locus with high LD having SNPs and two IgG glycan traits that are both associated with GD and the abundance of secreted plasma Desmoglein-2 in plasma. However, no direct association of AITD status with the abundance of secreted plasma Desmoglein-2. We previously performed glycome-wide association studies of AITD and TPOAb levels [4]. Genome-wide association studies of AITD and TPOAb-positivity were previously performed, and the findings are available via GWAS catalog [30] whereas pQTLs come from INTERVAL project[31]. IGP8 = the percentage of FA2[3]G1 glycan in total IgG glycans. IGP63 = The percentage of fucosylation (without bisecting GlcNAc) of agalactosylated structures.
